# Muscarinic receptors participation in angiogenic response induced by macrophages from mammary adenocarcinoma-bearing mice

**DOI:** 10.1186/bcr1005

**Published:** 2005-03-04

**Authors:** Eulalia de la Torre, Lilia Davel, María A Jasnis, Tomomi Gotoh, Eugenia Sacerdote de Lustig, María E Sales

**Affiliations:** 1 Departamento de Inmunobiología, Instituto de Oncología A.H. Roffo, Universidad de Buenos Aires, Buenos Aires, Argentina; 2 Department of Molecular Genetics, School of Medicine, Kumamoto University, Kumamoto, Japan

## Abstract

**Introduction:**

The role of macrophages in tumor progression has generated contradictory evidence. We had previously demonstrated the ability of peritoneal macrophages from LMM3 murine mammary adenocarcinoma-bearing mice (TMps) to increase the angiogenicity of LMM3 tumor cells, mainly through polyamine synthesis. Here we investigate the ability of the parasympathetic nervous system to modulate angiogenesis induced by TMps through the activation of the muscarinic acetylcholine receptor (mAchR).

**Methods:**

Peritoneal macrophages from female BALB/c mice bearing a 7-day LMM3 tumor were inoculated intradermally (3 × 10^5 ^cells per site) into syngeneic mice. Before inoculation, TMps were stimulated with the muscarinic agonist carbachol in the absence or presence of different muscarinic antagonists or enzyme inhibitors. Angiogenesis was evaluated by counting vessels per square millimeter of skin. The expression of mAchR, arginase and cyclo-oxygenase (COX) isoforms was analyzed by Western blotting. Arginase and COX activities were evaluated by urea and prostaglandin E_2 _(PGE_2_) production, respectively.

**Results:**

TMps, which stimulate neovascularization, express functional mAchR, because carbachol-treated TMps potently increased new blood vessels formation. This response was completely blocked by preincubating TMps with pirenzepine and 4-diphenylacetoxy-*N*-methylpiperidine (4-DAMP), M_1 _and M_3 _receptor antagonists, and partly by the M_2 _receptor antagonist methoctramine. M_1 _receptor activation by carbachol in TMps triggers neovascularization through arginase products because *N*^ω^-hydroxy-L-arginine reversed the agonist action. Preincubation of TMps with methoctramine partly prevented carbachol-stimulated urea formation. In addition, COX-derived liberation of PGE_2 _is responsible for the promotion of TMps angiogenic activity by M_3 _receptor. We also detected a higher expression of vascular endothelial growth factor (VEGF) in TMps than in macrophages from normal mice. Carbachol significantly increased VEGF expression in TMps, and this effect was totally reversed by methoctramine and pirenzepine. Arginase and COX inhibitors partly decreased VEGF derived from TMps.

**Conclusion:**

TMps themselves induce a potent angiogenic response that is augmented by carbachol action. mAchR activation triggers arginine metabolism, PGE_2 _synthesis and VEGF production, promoting neovascularization.

## Introduction

Malignant tumors contain macrophages (Mps) as a major component of the host leukocytic infiltrate, and the role of Mps in tumor progression has generated contradictory evidence [1]. It has been recognized that Mps can act either as negative regulators by achieving tumor cytotoxicity or as positive regulators by promoting tumor growth. Neovascularization, an essential step in tumor progression and metastasis development, can be modulated by the presence of Mps in the tumor microenvironment. Angiogenic stimuli can proceed from tumor cells and/or immune cells such as lymphocytes and Mps. We have previously demonstrated the ability Mps from tumor-bearing mice to exacerbate the angiogenic response elicited by LMM3 tumor cells (derived from a murine mammary adenocarcinoma), confirmed by CD31 positivity at the angiogenic site [2]. There are several molecules, such as nitrogen metabolites, prostaglandins, vascular endothelial growth factor (VEGF), fibroblast growth factor and placental growth factor, that exert proangiogenic functions [3]. Less knowledge is available about the autonomic regulation of tumor neovascularization. Here we investigate the role of the parasympathetic nervous system on the angiogenic activity exerted by peritoneal Mps from 7-day LMM3 mammary-tumor-bearing mice (TMps) by studying the expression and function of muscarinic acetylcholine receptors (mAchRs) in new blood vessel formation induced by TMps.

## Materials and methods

### Animals and tumor cell line

BALB/c mice (females 8 to 12 weeks old) from our Animal Care Division were used. Animal care was provided in accordance with the procedure outlined in the *Guide for Care and Use of Laboratory Animals *(NIH, 1986 edition). The tumor cell line LMM3 had previously been obtained from a spontaneous syngeneic mammary adenocarcinoma MM3 [4]. LMM3 cells were maintained as monolayers at 37°C in 5% CO_2 _in MEM supplemented with 5% FCS. Cells were detached with trypsin; only cell suspensions with more than 90% viability (assessed by Trypan blue) were used. Tumor-bearing mice were obtained by subcutaneous inoculation into the flank of 4 × 10^5 ^LMM3 cells.

### Purification of peritoneal macrophages

Resident peritoneal cells from normal mice and tumor-bearing mice were obtained by washing the peritoneal cavity previously inoculated with 5 ml of MEM supplemented with 10% FCS. The adherent Mps population from normal mice (NMps) and from 7-day tumor-bearing mice (TMps) were purified by adhesion to plastic for 2 hours. After being washed twice with PBS, adherent cells were scraped and resuspended in culture medium. Cell viability was assessed by the Trypan blue exclusion test; only suspensions with more than 95% viability were used.

### Angiogenesis assay

Mps and tumor cell-induced angiogenesis was quantified with an *in vivo *bioassay described previously [5]. In brief, tumor cell suspensions were prepared by detaching and washing LMM3 cells twice with fresh MEM. Cell concentration was adjusted to 3 × 10^6^cells/ml and female normal mice were inoculated intradermally in both flanks with 3 × 10^5 ^LMM3 cells, NMps or TMps in a total volume of 0.1 ml of MEM with a drop of Trypan blue to localize the site of inoculation. Before inoculation, Mps were treated for 1 hour with carbachol (100 nM) in the absence or presence of 1 μM atropine, 1 μM pirenzepine, 1 μM methoctramine, 1 μM 4-diphenylacetoxy-*N*-methylpiperidine (4-DAMP), 100 μM *N*^ω^-hydroxy-L-arginine (NOHA), 1 μM indomethacin or 10 μM NS-398. Cells were washed before inoculation. On day 5, animals were killed with ether, the skin was carefully separated from the underlying tissues and the vascular response was observed with a dissecting microscope (Wild) at × 6.4 magnification. The inoculated sites were photographed and the slides were projected on a reticular screen to count the number of vessels per mm^2 ^of skin. Angiogenesis was quantified as vessel density (δ), calculated as the total number of vessels divided by the total number of squares.

## Detection of muscarinic acetylcholine receptor subtypes by Western blotting

Purified Mps (10^6 ^cells) were lysed at 4°C with 0.5 ml of 0.5% Nonidet P40, 10 mM Tris-HCl, pH 7.4, 5 mM MgCl_2_, 50 mM NaCl, 1 mM EDTA, 1 mM EGTA, 50 mM NaF, 0.1 mM orthovanadate and the following protease inhibitors: 10 μg/ml aprotinin, 10 μg/ml leupeptin, 5 mM PMSF and 50 μg/ml soybean trypsin inhibitor. Lysates were sonicated for 30 s and centrifuged at 3,000 r.p.m. for 10 min at 4°C. Supernatants were centrifuged at 10,000 r.p.m. for 20 min at 4°C. The resulting supernatants were stored at -80°C. Protein concentration was determined by the Lowry method [6].

Samples were subjected to 7.5% SDS-PAGE minigel electrophoresis, with 30 μg of protein in each lane. Standards of known molecular masses were also seeded. After electrophoresis, proteins were transferred to a nitrocellulose membrane (Bio-Rad) and washed with distilled water. The nitrocellulose strips were blocked in buffer (20 mM Tris-HCl, 500 mM NaCl, 0.05% Tween 20 (TBST) with 5% skimmed milk) for 1 hour at 20 to -25°C and subsequently incubated overnight with goat anti-M_1_, anti-M_2 _and anti-M_3 _polyclonal antibodies (Santa Cruz Biotechnology) diluted 1:100 in TBST. After several rinses with TBST, strips were incubated with the second antibody (goat anti-mouse IgG conjugated with alkaline phosphatase, diluted 1:4,000 in TBST) at 37°C for 1 hour. Bands were revealed with a mixture of nitro blue tetrazolium chloride and 5-bromo-4-chloroindol-3-yl phosphate *p*-toluidine salt (NBT/BCIP) [7]. Quantification of the bands was performed with a computerized densitometer connected to an image analyzer (Bio-Rad GS700) and is expressed in optical density units per mm^2^.

## Arginase activity assay

Arginase activity was determined in cell lysates in accordance with methods described previously [8]. In brief, 10^5 ^cells were treated or not with 100 nM carbachol in the absence or presence of 100 μM NOHA, 1 μM atropine, 1 μM pirenzepine, 1 μM methoctramine or 1 μM 4-DAMP. After being washed, cells were lysed with 0.5 ml of 0.1% Triton X-100, 25 mM Tris-HCl, pH 7.4, containing 5 mM MnCl_2_. The enzyme was then activated by being heated at 56°C for 10 min. Arginine hydrolysis was performed by incubating 25 μl of the activated lysate with 25 μl of 0.5 M arginine, pH 9.7, at 37°C for 60 min. The reaction was stopped in acid medium. Urea concentration was measured at 540 nm with a microplate reader. Results are expressed as micromoles of urea per hour per million cells.

### Detection of arginase isoforms by Western blotting

Mps were rinsed twice with ice-cold PBS and then scraped into 300 μl lysis buffer (50 mM Tris-HCl, pH 7.5, 0.1 mM EDTA, 0.1 mM EGTA, 1 μg/ml leupeptin, 1 μg/ml aprotinin and 0.1 mM PMSF). Lysis was completed by sonication. Samples (25 μg) were subjected to 10% SDS-PAGE as described previously [9-11]. Nitrocellulose membranes were incubated overnight with a monoclonal anti-mouse arginase I antibody (BD Transduction Laboratories) or with a rabbit anti-arginase II antibody (a gift from Dr Masataka Mori). The secondary antibody anti-mouse or anti-rabbit IgG conjugated with alkaline phosphatase was added for 1 hour at 37°C. Proteins were revealed with NBT/BCIP and quantified by a densitometric analysis.

### Prostaglandin E_2 _assay

Prostaglandin E_2 _(PGE_2_) production by Mps was determined by RIA as described previously [12]. Purified Mps (2 × 10^6 ^cells per sample) were incubated for 1 hour at 37°C in a Dubnoff bath with carbogen in 1 ml of MEM with or without 100 nM carbachol in the absence or presence of 1 μM atropine, 1 μM methoctramine or 1 μM 4-DAMP, 1 μM indomethacin or 10 μM NS-398. After incubation, cells were centrifuged for 10 min at 200 *g *and supernatants were frozen at -80°C until the assay was performed. For PGE_2 _RIA, 100 μl samples or standards were incubated for 30 min with 500 μl of rabbit anti-PGE_2 _antiserum (Sigma) at 4°C. Then 5 pg of [^3^H]PGE_2 _(specific radioactivity 154 Ci/mmol; New England Nuclear) was added to each tube. All dilutions were performed in 0.01 M PBS, pH 7.4, containing 0.1% BSA and 0.1% sodium azide. After incubation, a dextran-coated charcoal suspension was added to separate the bound and free fractions. The supernatants were removed from each tube and scintillation solution (Optiphase Hisafe 3; Wallac) was added to determine the amount of radioactivity present. Results are expressed in picograms per 10^5 ^cells.

### Detection of cyclo-oxygenase (COX) isoforms by Western blotting

Purified Mps were washed twice in cold PBS and then resuspended in 300 μl of lysis buffer (20 mM Tris-HCl, 1 mM EDTA, 10 μg/ml leupeptin, 2 μg/ml aprotinin, 10 μg/ml dithiotreitol, 100 μg/ml soybean trypsin inhibitor, 1 mg/ml benzamidine). After 1 hour, lysates were centrifuged at 5,000 r.p.m. for 10 min. The resulting supernatants were stored at -80°C. Protein concentration was determined by the Lowry method [6].

Samples were subjected to 7.5% SDS-PAGE minigel electrophoresis, with 30 μg of protein in each lane. Standards of known molecular masses were also seeded. After electrophoresis, proteins were transferred to a nitrocellulose membrane (Bio-Rad) at 4°C for 18 hours. Membranes were then washed with distilled water and incubated with blocking solution (5% skimmed milk in TBST) for 1 hour at 20 to -25°C. Membranes were incubated with rabbit polyclonal anti-COX-1 or anti-COX-2 antibodies (Cayman Chemical) in Tris-buffered saline for 90 min at room temperature. Then secondary anti-rabbit IgG antibody conjugated with alkaline phosphatase was added for 1 hour at 37°C. Proteins were revealed with NBT/BCIP and quantified by a densitometric analysis [13].

### Detection of VEGF by Western blotting

Production of VEGF was measured in lysates from untreated TMps or TMps treated with 100 nM carbachol for 1 hour in the absence or presence of 1 μM atropine, 1 μM pirenzepine, 1 μM methoctramine or 1 μM 4-DAMP or the enzyme inhibitors 100 μM NOHA, 1 μM indomethacin or 10 μM NS-398. Cells were then cultured without FCS at 37°C for 24 hours in 100 mm Petri dishes. After being washed twice with cold PBS, TMps were lysed in 10 mM Tris-HCl, pH 8, 1% Triton X-100, 100 mM NaCl, 10 mM EGTA, 10 mM EDTA, with protease inhibitors (1 mM PMSF, 10 μg/ml leupeptin, 10 μg/ml aprotinin). After 1 hour in an ice bath, lysates were centrifuged at 10,000 r.p.m. for 10 min at 4°C. Samples were subjected to 10% SDS-PAGE electrophoresis. Proteins were transferred to nitrocellulose membranes and, after several rinses with doubly distilled water, were blocked with 5% skimmed fat milk in TBST buffer. The primary antibody (goat polyclonal anti-VEGF; Santa Cruz Biotechnology) was added for 18 hours, and the secondary antibody anti-goat IgG conjugated with alkaline phosphatase was added for 1 hour at 37°C. Proteins were detected with NBT/BCIP and quantified by densitometric analysis [13,14].

### Drugs

All drugs were purchased from Sigma-Aldrich unless otherwise stated. Solutions were prepared fresh daily.

### Statistics

Results are given as means ± SEM for at least three independent experiments. The statistical significance of differences between groups was analyzed by analysis of variance, Tukey's modified *t*-test or the Mann–Whitney test, using the STAT PRIMER program; *P *< 0.05 was considered to be statistically significant.

## Results

### Carbachol stimulates angiogenesis induced by TMps

We have reported previously that a small number of TMps (2 × 10^3 ^to 2 × 10^4 ^cells) from 7-day LMM3 tumor-bearing mice were unable to induce an angiogenic response in syngeneic mice. The vessel density (δ) of mice inoculated with TMps (δ = 1.70 ± 0.15) was not significantly different from that observed in normal skin (δ = 1.65 ± 0.20). In larger numbers (3 × 10^5^), TMps elicit positive angiogenesis (δ = 2.84 ± 0.06), similar to that observed with the same number of LMM3 tumor cells (δ = 2.91 ± 0.38). The addition of 100 nM carbachol increased the neovascularization induced by TMps by 112% (Table 1). The participation of mAchR was confirmed by blunting the carbachol action with 1 μM atropine. Peritoneal NMps were unable to induce angiogenesis: the neovascular response (δ = 1.86 ± 0.55) was similar to that observed in normal skin. The addition of carbachol, at the same dose, did not modify the density of blood vessels (δ = 1.80 ± 0.46).

We then investigated the participation of mAchR subtypes in neovascularization induced by TMps. As shown in Table 1, blockade of M_1 _or M_3 _receptors with 1 μM pirenzepine or 4-DAMP, respectively, completely abolished the stimulatory effect of carbachol on angiogenesis, whereas preincubation with 1 μM methoctramine (an M_2 _antagonist) partly prevented the carbachol action.

### Arginase and COX products are involved in angiogenesis induced by TMps

Table 2 summarizes results indicating that preincubating TMps with 100 μM NOHA, 1 μM indomethacin or 10 μM NS-398 significantly blunted carbachol-stimulated angiogenesis, demonstrating the participation of arginase and COX in this effect.

**Figure 1:**
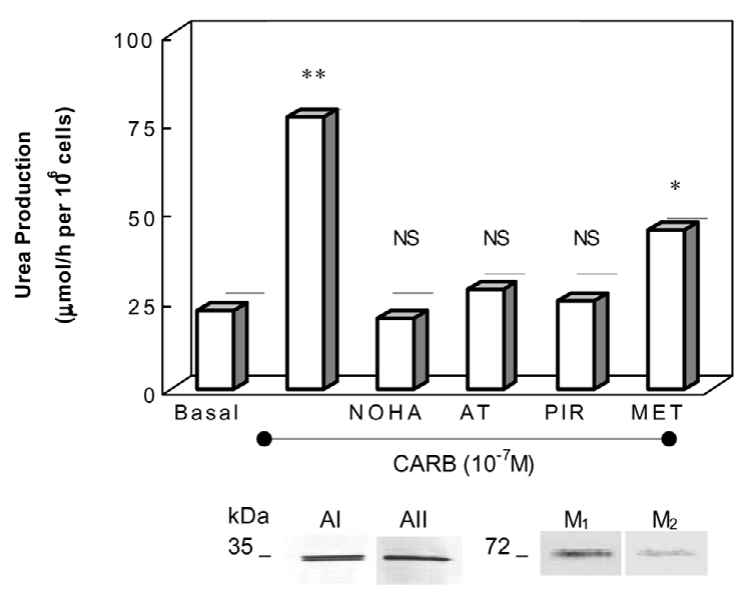
Arginase and muscarinic receptors in TMps. Upper panel: macrophages from 7-day LMM3 tumor-bearing mice (TMps) were treated with carbachol (CARB) (100 nM) in the absence or presence of 100 μM *N*^ω^-hydroxy-L-arginine (NOHA), 1 μM atropine (AT), pirenzepine (PIR) or methoctramine (MET). Arginase activity was measured by urea production as μmol/h per 10^6 ^cells. Values are means ± SEM for five experiments. **P < 0.001, *P < 0.05 compared with basal; NS, not significantly different from basal by Tukey's modified *t*-test. Lower panel: Western blot assay to detect arginase isoforms and muscarinic receptors (M) in lysates of TMps. The molecular masses of the bands indicated on the left are coincident with arginase I, II and M_1 _and M_2 _proteins. One representative experiment of three is shown.

We have previously reported that arginase I and II were constitutively expressed in Mps, and their expression and activity were upregulated in TMps in comparison with NMps. Our present results indicate that carbachol, at the same dose that triggers neovascularization, increases urea formation (Fig. 1). This overproduction was completely reversed by NOHA (100 μM), an enzyme-specific inhibitor of arginase. mAchR activation was also blunted by 1 μM atropine or with 1 μM pirenzepine, indicating a relation between M_1 _receptor subtype activation and arginase as its effector enzyme. It is also shown in Fig. 1 that the M_2 _selective antagonist methoctramine partly blunted the action of carbachol. Preincubation of TMps with 4-DAMP did not modify the action of carbachol on urea formation (78 ± 7 μmol/h per 10^6 ^cells). Western blotting therefore shows the presence of M_1 _and M_2 _receptor proteins in the membrane-enriched fraction of TMps (Fig. 1).

**Figure 2:**
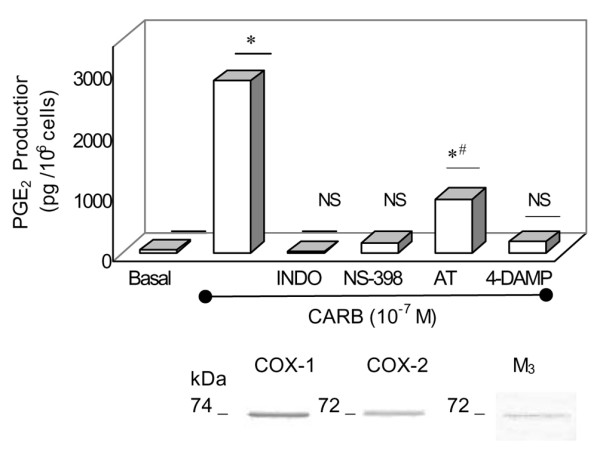
Cyclo-oxygenase (COX) muscarinic receptors in TMps. Upper panel: macrophages from 7-day LMM3 tumor-bearing mice (TMps) were treated with carbachol (CARB) (100 nM) in the absence or presence of 1 μM indomethacin (INDO) 10 μM NS-398, 1 μM atropine (AT) or 4-DAMP. COX activity was measured by prostaglandin E_2 _(PGE_2_) liberation by TMps as pg/10^6 ^cells. Values are means ± SEM for three experiments. *P < 0.001 compared with basal; ^#^*P *< 0.001 compared with CARB; NS, not significantly different from basal by Tukey's modified *t*-test. Lower panel: Western blot assay to detect COX isoforms and muscarinic receptors (M) in lysates of TMps. Molecular masses of the bands indicated on the left are coincident with COX-1, COX-2 and M_3 _proteins. One representative experiment of three is shown.

Prostaglandins are important mediators in tumor progression because they promote tumor growth and immunosuppress tumor hosts. Here we show that 100 nM carbachol markedly increased the liberation of PGE_2 _by TMps (Fig. 2). This stimulatory action was inhibited by preincubating cells with 1 μM indomethacin or with 10 μM NS-398, a non-selective COX inhibitor and a COX-2-selective inhibitor, respectively. In addition, we observed that M_3 _receptor subtype is involved in carbachol-induced PGE_2 _liberation: not only did 1 μM atropine blunt the agonist action, but a 1 μM 4-DAMP blockade was also effective (Fig. 2). Neither pirenzepine (2,879 ± 181 pg of PGE_2_/10^6 ^cells) nor methoctramine (2,799 ± 197 pg PGE_2_/10^6 ^cells) modified carbachol-induced PGE_2 _liberation. We also detected M_3 _receptor subtype expression in the TMps membrane-enriched fraction by Western blotting (Fig. 2).

### Participation of VEGF in angiogenesis induced by TMps

**Figure 3:**
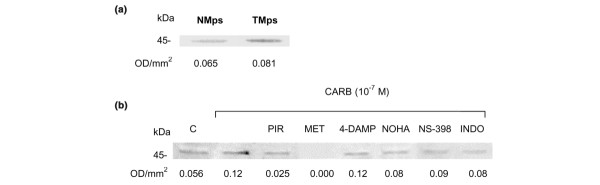
Western blot assay to detect vascular endothelial growth factor (VEGF) expression. **(a) **Expression in normal macrophages (NMps) and macrophages from 7-day LMM3 tumor-bearing mice (TMps). **(b) **TMps were treated with carbachol (CARB) (100 nM) in the absence or presence of 1 μM pirenzepine (PIR), methoctramine (MET) or 4-DAMP. Lane C, control (TMps without treatment). Bands were quantified in optical density units per square millimeter (OD/mm^2^). The molecular mass indicated at the left corresponds to the VEGF isoform detected. One representative experiment of three is shown.

Several growth factors and/or cytokines are involved in tumor angiogenesis. VEGF is the most extensively studied. Here we measured VEGF expression in lysates of Mps. Western blot experiments indicate that TMps express larger amounts of VEGF than NMps do (Fig. 3a). In addition, carbachol significantly increases VEGF derived from TMps. Preincubation of cells with 1 μM methoctramine or pirenzepine blocked the action of carbachol on VEGF expression, whereas 1 μM 4-DAMP was ineffective in preventing agonist action. When TMps were treated with 100 μM NOHA, 10 μM NS-398 or 1 μM indomethacin, VEGF protein expression was decreased by almost 30% (Fig. 3b).

## Discussion

Mps perform multiple functions that are essential in tissue remodeling, wound healing, inflammation and immunity. These cells form the major component of the mononuclear leukocyte population of some solid tumors [1,15]. In the 1980s, Polverini and Leibovich demonstrated that tumor-associated Mps isolated from 3-methycholanthrene-induced rat fibrosarcoma were potent stimulators of *in vivo *neovascularization and bovine endothelial cell proliferation; depletion of Mps from tumor cell suspensions significantly decreased their angiogenic potential, suggesting that neovascularization was mediated in part by Mps [16].

Taking into account the fact that murine mammary adenocarcinomas arising spontaneously in BABL/c mice in our laboratory are poorly infiltrated by Mps, we showed that peritoneal Mps from 7-day tumor-bearing mice, when present at low concentrations, contribute to the enhancement of LMM3 angiogenesis by providing polyamine precursors to tumor cells [2]. Although the origin of tumor-infiltrating Mps has been discussed extensively, evidence supports both recruitment from the circulating pool of monocytes and the proliferation of the local Mps population, and it has recently been discussed that Mps could become angiogenic in the presence of diverse stimuli such as growth factors or low oxygen tension as well as soluble tumor antigens [17,18]. Here we show that peritoneal TMps, when inoculated in a number equal to that of LMM3 tumor cells, themselves elicit a potent angiogenic response. In contrast, 'unstimulated' NMps did not promote angiogenesis in our model. Further investigation is required to determine whether TMps activation occurs in the host-tumor interface or can be triggered at distance by soluble cytokines and/or tumor antigens.

Other authors have shown that the levels and functions of lymphocytes, granulocytes, Mps and natural killer cells are under the regulation of the autonomic nervous system [19]. We showed that the activation of mAchR in TMps by the cholinergic agonist carbachol increases their angiogenic ability. The participation of muscarinic receptors was demonstrated by preincubating cells with the non-selective muscarinic antagonist atropine. Angiogenesis is now considered an important step during inflammation and cancer, and it might be necessary as a local, protective response against invasion by pathogens and the proliferation of transformed cells. It is also important in tumor growth and metastasis. The nervous system reflexively regulates the inflammatory response and it has been recently documented that acetylcholine, the principal vagal neurotransmitter, significantly attenuates the release of cytokines (tumor necrosis factor, IL-1, IL-6 and IL-18, but not the anti-inflammatory cytokine IL-10) in lipopolysaccharide-stimulated human macrophage cultures [20]. These anti-inflammatory actions are generally related to nicotinic receptor stimulation [21]. In our model, mAchR stimulation seems to be promoting pro-inflammatory actions by stimulating angiogenesis induced by TMps. It remains to be tested whether the activation of nicotinic receptors in TMps might be exerting anti-inflammatory actions.

M_1_, M_2 _and M_3 _antagonists decreased the carbachol stimulation of neovascularization induced by TMps, showing a collaborative activation of different mAchR subtypes in the neovascular response. It has recently been documented that different mAchR activation controls different functions in distinct systems simultaneously. The activation of M_1 _and M_3 _receptors by carbachol induces pigment granule dispersion in isolated retinal pigment epithelium from bluegill. Carbachol-induced pigment granule dispersion is blocked by the muscarinic antagonist atropine, by the M_1 _antagonist pirenzepine and by the M_3 _antagonist 4-DAMP [22]. We also showed that the activation of M_1_, M_2 _and M_3 _receptors by carbachol is involved in the proliferation of two different murine mammary adenocarcinoma cell lines, LM3 and LM2 [23].

The carbachol stimulation of angiogenesis induced by TMps occurs by the signaling of M_1 _to arginase, because pirenzepine totally blocked the carbachol stimulation of urea production. Arginase I and II are overexpressed in TMps in comparison with NMps and are involved in the positive modulation by TMps of angiogenesis induced by LMM3 mammary tumor cells [2]. We were the first to report that carbachol was able to stimulate the proliferation of tumor cells by arginine metabolism through arginase linked to M_1 _receptors in LM2 cells, derived from M2 murine mammary adenocarcinoma [23].

We also observed that methoctramine partly blunted carbachol-stimulated vascularization and urea formation, indicating that M_2 _receptors are also involved in this effect. We have previously documented a collaborative action of M_2 _and M_3 _receptor activation by carbachol, which increases amylase secretion in lipopolysaccharide-inflamed salivary glands by stimulating PGE_2 _liberation [7]. We are therefore reporting that the expression and function of M_1 _and M_2 _receptors are involved in the control of angiogenesis induced by TMps, by stimulating polyamine synthesis in these cells.

The tumor microenvironment is rich in inflammatory cytokines, growth factors and chemokines, but generally poor in cytokines associated with a sustained immune antitumor response. It is now accepted that tumor-associated Mps produce soluble mediators that contribute to tumor progression. Our results indicate that the parasympathetic nervous system positively modulates neovascularization induced by TMps by stimulating M_3 _receptors and PGE_2 _liberation. Because indomethacin and NS-398 blunted the carbachol action on PGE_2 _synthesis, the COX-1 and COX-2 isoenzymes are involved in angiogenesis induced by TMps. In particular, COX-2 protein expression is highly upregulated in TMps in comparison with NMps (data not shown). Several authors have stated that there is a role of COX-2 expression and function not only in tumors but also in immune cells from the host [14,18,25]. The overproduction of this prostanoid could be responsible for an autocrine loop that also promotes immunosuppression of the host.

Previous results indicate that activation of G-protein-coupled receptors encoded by Kaposi's sarcoma-associated herpesvirus could be increasing VEGF expression and promoting an angiogenic response that characterizes Kaposi's sarcoma lesions [26]. In support of this view, we observed that stimulation of mAchR in TMps by carbachol increased the 45 kDa isoform of VEGF. This effect is linked to activation of the M_2 _and M_1 _receptors, which in turn promote the arginine metabolic pathway through arginase. We have previously observed that the arginase pathway is involved in the angiogenic response induced by LMM3 cells derived from a murine mammary adenocarcinoma. These cells, which exert a potent angiogenic response quantitatively similar to that induced by TMps, also produce significant amounts of VEGF [13]. Our results show that VEGF production by TMps depends partly on arginase metabolism because NOHA decreases VEGF production. Pretreatment of cells with COX inhibitors also diminished VEGF derived from TMps. In this way, the expression of COX-1 and COX-2 and their product PGE_2 _has been shown to be promoters of angiogenesis by modulating the synthesis of various factors, including VEGF [13,27]. It must be taken into account that the stimulation of VEGF expression by COX-derived PGE_2 _in TMps is independent of M_3 _receptor activation.

## Conclusion

Here we propose a new mechanism involved in angiogenesis induced by peritoneal TMps through mAchR activation sustained by the formation of arginase products and PGE_2_, which could be acting as promoters of the stimulation by VEGF of endothelial cell proliferation, vessel sprouting and organization during tumor progression or metastasis.

## Abbreviations

COX = cyclo-oxygenase; 4-DAMP = 4-diphenylacetoxy-*N*-methylpiperidine; FCS = fetal calf serum; IL = interleukin; kDa = kilodaltons; mAchR = muscarinic acetylcholine receptor; MEM = minimal essential medium; Mps = macrophages; NBT/BCIP = nitro blue tetrazolium/5-bromo-4-chloroindol-3-yl phosphate; NMps = peritoneal macrophages from normal mice; NOHA = *N*^ω^-hydroxy-L-arginine; PBS = phosphate-buffered saline; PGE_2 _= prostaglandin E_2_; RIA = radio-immunoassay; TBST = Tris-buffered saline containing Tween 20; TMps = peritoneal macrophages from LMM3 murine mammary adenocarcinoma-bearing mice; VEGF = vascular endothelial growth factor.

## Competing interests

The author(s) declare that they have no competing interests.

## Authors' contributions

E de la T performed the Western blot assays, LD performed the *in vivo *angiogenesis experiments and the statistical analysis, MAJ made helpful criticism in discussion, TG developed the anti-arginase II antibody, ESL participated in the study design and coordination, and MES performed RIAs and conceived of the study, and participated in its design and coordination. All authors read and approved the final manuscript.

## Figures and Tables

**Table 1:** Participation of mAchR subtypes in angiogenesis induced by TMps

Treatment	Angiogenic response (vessels/mm^2^)	*n*
None	2.33 ± 0.07	5
CARB	4.98 ± 0.40**	5
AT + CARB	2.57 ± 0.25	5
PIR + CARB	2.49 ± 0.63	4
MET + CARB	3.35 ± 0.17*	5
4-DAMP + CARB	2.64 ± 0.48	6

Peritoneal macrophages (3 × 10^5^) from 7-day LMM3 tumor-bearing mice (TMps) untreated or stimulated with carbachol (CARB; 100 nM) in the absence or presence of 1 μM atropine (AT), pirenzepine (PIR), methoctramine (MET) or 4-diphenylacetoxy-*N*-methylpiperidine (4-DAMP) were inoculated intradermally into normal mice to evaluate the angiogenic response. **P < 0.0001, *P < 0.05 compared with untreated TMps (Mann–Whitney test). *n*, number of sites evaluated. Results are means ± SEM for three experiments.

**Table 2:** Participation of arginase and COX in angiogenesis induced by TMps

Treatment	Angiogenic response (vessels/mm^2^)	*n*
None	2.34 ± 0.06	5
CARB	4.96 ± 0.41*	5
NOHA + CARB	2.4 ± 0.6	4
INDO + CARB	1.9 ± 0.92	8
NS-398 + CARB	1.3 ± 0.31	5

Peritoneal macrophages (3 × 10^5^) from 7-day LMM3 tumor-bearing mice (TMps) stimulated with carbachol (CARB; 100 nM) in the absence or presence of *N*^ω^-hydroxy-L-arginine (NOHA; 100 μM), indomethacin (INDO; 1 μM) or NS-398 (10 μM) were inoculated intradermally into normal mice to evaluate the angiogenic response. *P < 0.001 compared with control (without treatment) (Mann–Whitney test). *n*, number of sites evaluated. Results are means ± SEM for three experiments.
